# Editorial: Targeting signalling pathways in inflammatory diseases

**DOI:** 10.3389/fimmu.2023.1241440

**Published:** 2023-08-01

**Authors:** Mirza S. Baig, Teresa L. M. Thurston, Rahul Sharma, Rajat Atre, Uzma Saqib, Rakhi Khabiya, Shreya Bharti, Chit L. Poh

**Affiliations:** ^1^ Department of Biosciences and Biomedical Engineering (BSBE), Indian Institute of Technology Indore (IITI), Indore, India; ^2^ Centre for Bacterial Resistance Biology, Imperial College London, London, United Kingdom; ^3^ School of Life Sciences, Devi Ahilya Vishwavidyalaya (DAVV), Indore, India; ^4^ Centre for Virus and Vaccine Research, Sunway University, Bandar Sunway, Malaysia

**Keywords:** chronic inflammation, signalling pathways, inflammatory response, macrophages, MAL (TIR domain-containing adaptor protein)

Chronic inflammation, characterized by a persistent elevation of circulating pro-inflammatory cytokines, is associated with the pathogenesis of many non-communicable diseases that cause a worldwide health burden and a reduction in quality of life (1). The identification of possible therapeutic targets implicated in the regulation of inflammation offers the opportunity to limit the dangers associated with an imbalance in the inflammatory response (2). Adaptor proteins represent key signaling molecules that regulate the host’s innate immune response to infections, acting as links between receptors and other molecules in several signaling cascades (3, 4). The evident importance of these proteins in the pathophysiology of different chronic inflammatory illnesses makes them attractive therapeutic targets (4).

Here, we focus on a crucial inflammation-related adaptor of Toll-like receptors (TLR), called MyD88 adaptor-like (MAL) or Toll-interleukin-1 Receptor (TIR) domain-containing adaptor protein (TIRAP). MAL contains a TIR domain, required for mediating interactions with receptors on the membrane and with downstream signaling molecules (5). MAL represents a key mediator of TLR signaling in immune cells such as macrophages (6, 7), where activation of TLR2 and TLR4 cause persistent inflammation in a MAL-dependent fashion (7). Following receptor-mediated detection of pathogenic ligands, MAL mediates various protein-protein interactions (**Figure 1A**).

**Figure 1:**
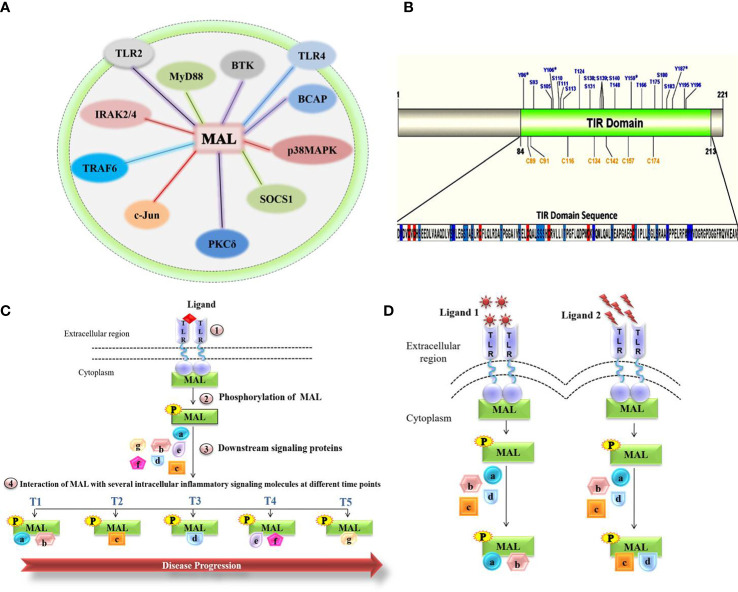
Molecular role of MAL in chronic inflammatory diseases. **(A)** Representation of the total network of TIRAP protein-protein interactions in macrophage inflammatory signaling. **(B)** Representation of the MAL-TIR domain and computational prediction of phosphorylation and S-nitrosylation (SNO) positions. **(C)** Schematic highlighting the interactions of MAL involved in disease progression. **(D)** MAL interactions under various stimulants.

Tyrosine kinases, including BTK and PKCδ, have a major role in the activation of MAL, with BTK mediating phosphorylation on the four MAL residues Y86, Y106, Y159, and Y187 (5), as well as PKCδ phosphorylating Y86 and Y106 in MAL’s TIR domain (8). The overlapping phosphorylation sites highlight the possible interconnected activities of these kinases with MAL, as well as pointing to possible context-dependent fine-tuning of MAL activity (8). After activation, MAL interacts with critical inflammatory proteins and eventually activates several transcriptional factors involved in the release of pro-inflammatory cytokines, which consequently leads to an inflammatory response (5). Contrary to phosphorylation, nitric oxide (NO)-mediated S-nitrosylation of cysteine residues in MAL’s TIR domain attenuates the inflammatory response, which may be due to MAL interactions with downstream inflammatory signaling molecules (9).

Upon TLR4 activation, the inflammatory response involves the activation of transcription factors such as NF-kB and AP1, thereby generating pro-inflammatory cytokines. Baig et al. reported the formation of a heterotrimeric complex of p38MAPK, PKCδ, and MAL in LPS- stimulated macrophages (10). This reiterates the potential role of MAL in regulating inflammatory pathways via various protein interactions (10, 11). On the basis that the MAL-PKCδ interaction is crucial in inflammatory signaling mediated by TLR2/4 (10) and that PKCδ phosphorylates the MAL TIR domain, Rajpoot et al. conducted a virtual screen of FDA-approved drugs that would disrupt the MAL-PKCδ interaction (12). This screen revealed dorzolamide (DZD) as a novel therapeutic, where it suppressed the PKCδ-MAL-p38 MAPK signaling axis to inhibit inflammation (12). A significant (42%) increment in survival was observed in DZD-treated mice as compared to LPS alone–injected mice, validating the abrogation of inflammatory response in drug-treated mice (12). MAL also interacts with c-Jun, a subunit of the AP-1 transcription factor complex that is activated upon LPS stimulation of TLR4 (13). The interaction of MAL with c-Jun resulted in the transactivation and translocation of c-Jun, which ultimately resulted in the production of proinflammatory cytokines (13), thus making the interaction between these two proteins a potential therapeutic target. Indeed, Mansi et al. proposed a repurposed anti-inflammatory drug Gefitinib that abrogated the interaction of MAL with c-Jun, thereby inhibiting the cell’s inflammatory response (13).

As post-translational modifications seem to be the major contributing factor toward MAL’s variable interactions and eventual inflammatory responses, we were interested to know all the potential phosphorylation and nitrosylation sites on the TIR domain (**Figure 1B**). Modifications at these sites may variably impact the interactions with known and unknown interaction partners, regulators, and downstream mediators. Likely, MAL’s interactions with kinases and other proteins vary temporally and spatially. Inadvertently, each of these interactions [**Figure 1A** and reviewed in detail by Rajpoot et al. (5)] represent potential points of therapeutic intervention. Thus, it remains crucial to understand how MAL is regulated and what interactions it forms under the influence of different stimulants acting on different TLRs. Once defined, the impact of individual interactions can then be determined during disease progression. Based on the studies published so far, we hypothesize (**Figures 1C, D**) that different MAL-mediated protein-protein interactions define the severity of chronic inflammation. In conclusion, unraveling the protein-protein interactions of MAL would not only lead us to a greater understanding of the underlying signaling mechanisms that occur in the progression of various life-threatening chronic inflammatory conditions, but would also direct us toward the development of important therapeutic strategies for disease treatment.

## Author contributions

Conceptualization and supervision: MSB; writing and editing: MB, TT, RS, RA, US, RK, SB, and CP. All authors contributed and approved the submitted version.
